# Association of Lower Alanine Transaminase (ALT) and Aspartate Aminotransferase (AST) Levels With Eyelid Edema in Graves' Orbitopathy: A Retrospective Cross-Sectional Study

**DOI:** 10.7759/cureus.90519

**Published:** 2025-08-19

**Authors:** Yuying Xiang, Rui Li, Shuang Wang, Guang Zhao, Fagang Jiang, Junjie Yang

**Affiliations:** 1 Department of Ophthalmology, Wuhan Union Hospital, Wuhan, CHN; 2 Department of Epidemiology and Public Health, Tongji Medical College, Huazhong University of Science and Technology, Wuhan, CHN

**Keywords:** alt, ast, eyelid edema, graves' orbitopathy, inflammation, liver enzymes

## Abstract

Background: Graves’ orbitopathy (GO) is a common autoimmune disorder affecting the eye muscles and orbital tissues, often leading to eyelid edema. The potential role of liver enzymes in this condition remains unclear.

Methods: We conducted a retrospective cross-sectional study on 303 GO patients from Wuhan Union Hospital between 2015 and 2025. Participants were grouped according to the presence of eyelid edema. Univariable and multivariable logistic regression models were applied to evaluate the associations between alanine transaminase (ALT), aspartate aminotransferase (AST), and eyelid edema, with adjustments for sex, age, and lymphocytes.

Results: Our analysis showed that lower ALT and AST levels were significantly associated with an increased likelihood of eyelid edema in the fully adjusted models (ALT per 10 U: OR = 0.813, 95% CI = 0.678-0.975; AST: OR = 0.681, 95% CI = 0.489-0.947). A linear dose-response relationship was observed: for each 10-unit decrease in ALT or AST, the observed prevalence of eyelid edema increased by 18.7%-31.9%.

Conclusion: This study demonstrated an association between liver enzymes (AST and ALT) and eyelid edema in GO patients. The findings suggest a potential link between liver function and the pathogenesis of GO-related edema. Further prospective studies are required to clarify the causal relationship, explore the underlying mechanisms, and assess the clinical utility of liver enzymes as biomarkers for GO severity or progression.

## Introduction

Graves’ orbitopathy (GO), also known as thyroid eye disease (TED) or thyroid-associated ophthalmopathy (TAO), is the most frequent form of extrathyroidal involvement in Graves’ disease. It is an autoimmune disorder characterized by the immune targeting of antigens shared between the thyroid gland and orbital tissues, leading to orbital disfigurement, proptosis, lid retraction, chemosis, and even visual loss [1]. While the pathogenesis of GO remains incompletely understood, emerging evidence highlights the interplay between thyroid autoantibodies, genetic polymorphisms (e.g., human leukocyte antigen [HLA], cytotoxic T-lymphocyte-associated protein 4 [CTLA-4]), and environmental triggers such as smoking [2,3]. Approximately 86.2% of GO patients present with hyperthyroidism, while 7.9% are euthyroid, highlighting the complexity of its clinical spectrum [4]. Notably, the female-to-male ratio in severe GO is 1:4, suggesting sex-specific factors in disease progression [1].

The Clinical Activity Score (CAS) provides a standardized framework for assessing GO severity, with eyelid edema being a key inflammatory marker [1]. Recent studies have further linked systemic inflammation to GO manifestations, such as the correlation between elevated neutrophil-to-lymphocyte ratio (NLR) and cholesterol dysregulation with disease activity, implying that systemic biomarkers may reflect orbital inflammation [5,6]. Importantly, liver enzymes such as alanine aminotransferase (ALT) and aspartate aminotransferase (AST), traditionally markers of hepatic injury, are increasingly recognized for their association with metabolic and inflammatory states [7]. Elevated ALT/AST levels directly correlate with insulin resistance in non-alcoholic fatty liver disease (NAFLD) through impaired hepatic lipid metabolism, while in alcoholic liver injury, they reflect amplified pro-inflammatory cytokine production (TNF-α, IL-6) and neutrophil infiltration [8]. These enzymes also serve as sentinels of mitochondrial dysfunction in metabolic syndrome, where disrupted NADH homeostasis triggers oxidative stress and activates NLRP3 inflammasome signaling [9]. Furthermore, their elevation in metabolic dysfunction-associated steatohepatitis (MASH) signifies synergistic interactions between dysregulated bile acid metabolism and Kupffer cell-mediated inflammation [10].

In Graves' disease, research on liver enzyme abnormalities has demonstrated that 60% of untreated patients exhibit at least one abnormal liver function indicator, with the ALT and AST reaching 33% and 23%, respectively [11]. These findings may be attributed to the direct cytotoxic effects of thyroid hormones on hepatocytes, liver injury induced by antithyroid drugs, and hepatocyte destruction mediated by autoimmune reactions.

Despite these advances, the role of liver enzymes in GO-related eyelid edema remains unexplored. This study aims to investigate the associations between ALT/AST levels and eyelid edema in GO patients, hypothesizing that liver enzyme dysregulation may reflect systemic inflammation or metabolic alterations contributing to orbital pathology. Importantly, exploring the role of ALT and AST in GO could potentially offer novel insights into the disease mechanisms and provide additional clinical markers for assessing disease activity and severity. While there are existing markers for GO in clinical practice, such as thyroid-stimulating hormone receptor antibodies (TSHR-Ab) [12], they may not fully capture the metabolic and inflammatory aspects of the disease. Incorporating liver enzyme levels as complementary markers could enhance the comprehensive evaluation of GO patients, aiding in more precise diagnosis, prognosis assessment, and therapeutic decision-making.

Future longitudinal studies are warranted to establish causality and explore the clinical utility of liver enzymes as biomarkers for GO severity or progression.

## Materials and methods

Study design and data collection

This retrospective cross-sectional study enrolled 303 patients diagnosed with Graves’ orbitopathy at Wuhan Union Hospital between January 2015 and January 2025. GO diagnosis followed the European Group on Graves’ Orbitopathy (EUGOGO) consensus criteria, which include clinical signs such as eyelid retraction, proptosis, and orbital inflammation. Inclusion criteria were (1) age ≥18 years; (2) meeting the above EUGOGO diagnostic criteria; and (3) availability of complete clinical and laboratory data, including ALT and AST levels. Exclusion criteria were (1) history of ocular surgery or trauma within 12 months; (2) glucocorticoid use within six months; (3) pregnancy, cancer, severe renal/liver disease (defined as ALT >100 U/L, or AST >100 U/L, or imaging-confirmed cirrhosis), or hematological disorders. Data were extracted from electronic medical records using the Solution EDC system (Shanghai, China).

Clinical and laboratory assessment

Clinical evaluation documented eyelid edema, conjunctival redness, and chemosis. Eyelid edema was defined as the presence of visible swelling of the eyelids, as recorded by the treating ophthalmologist during the clinical examination, based on the standardized clinical assessment guidelines recommended by the EUGOGO. The activity of GO was assessed using the CAS according to EUGOGO recommendations, with a score ≥ 3/7 indicating active disease.

Laboratory parameters included complete blood count, hepatorenal function (ALT, AST, creatinine), electrolytes, and thyroid hormones (TSH, FT3, FT4). Thyroid status was classified as euthyroidism (TSH: 0.4-4.0 mIU/L; FT3: 3.1-6.8 pmol/L; FT4: 12-22 pmol/L) or impaired function (outside these ranges). Two researchers independently verified data entries, with discrepancies resolved by a third investigator.

Statistical analysis

Continuous variables were expressed as medians (interquartile range) and compared using the Wilcoxon rank-sum test. Categorical variables were analyzed by the chi-square test. Variables associated with eyelid edema were initially identified through univariate logistic regression, with a significance threshold of p < 0.1 for inclusion in multivariable models. Multivariable logistic regression was performed to adjust for potential confounders, including sex, age, and lymphocyte count.

Additionally, the relationship between ALT and AST levels with eyelid edema was further examined using curve fitting analysis with generalized additive models in the "mgcv" package (R). Subgroup analyses were conducted to assess the association between eyelid edema and covariates across different subgroups.

All statistical analyses were performed using R 4.0.3 (R Foundation for Statistical Computing, Vienna, Austria) and EasyR (Solutions Inc., Shanghai, China). A two-sided p-value of < 0.05 was considered statistically significant.

Ethical approval

The study was approved by the Ethics Committee of Wuhan Union Hospital (Approval Number: 20211031) and registered at the Chinese Clinical Trial Registry (ChiCTR2200063429). Informed consent was waived due to the retrospective design.

## Results

Baseline characteristics

A total of 303 GO patients were enrolled in the study. The demographic and clinical characteristics are summarized in Table 1. The median age was 47 years (IQR: 37-54), with a balanced sex distribution (146 [48.2%] male, 157 [51.8%] female). The most prevalent ocular manifestations included conjunctival redness (164 [54.1%]) and eyelid edema (159 [52.5%]), while conjunctival chemosis (63 [20.8%]) and eyelid erythema (28 [9.2%]) were less frequently observed.

**Table 1:** Baseline characteristics of participants.

Variables	Proportion (n = 303)
Male	146 (48.2%)
Female	157 (51.8%)
Age, years	47.0 (37.0-54.0)
Without eyelid edema	144 (47.5%)
With eyelid edema	159 (52.5%)
Without redness of the eyelid	275 (90.8%)
With redness of the eyelid	28 (9.2%)
Without chemosis of the conjunctiva	240 (79.2%)
With chemosis of the conjunctiva	63 (20.8%)
Without redness of the conjunctiva	139 (45.9%)
With redness of the conjunctiva	164 (54.1%)

Comparison of patients with and without eyelid edema

Patients were stratified by the presence of eyelid edema (Table 2). No significant differences were observed in age, sex, or thyroid function between groups (all p > 0.05). However, ALT and AST levels were significantly lower in patients with eyelid edema (ALT: 19.0 vs. 21.0 U/L, p=0.033; AST: 18.0 vs. 20.0 U/L, p=0.032). Other hematological parameters (FT3, FT4, TSH) showed no significant difference between groups.

**Table 2:** Comparison of clinical and laboratory parameters between Graves’ disease patients without and with eyelid edema WBC: white blood cell; RBC: red blood cell; Hb: hemoglobin; MCV: mean corpuscular volume; MCHC: mean corpuscular hemoglobin concentration; TBIL: total bilirubin; ALT: alanine aminotransferase; AST: aspartate aminotransferase; AKP: alkaline phosphatase; GGT: glutamyl transpeptidase. Data were presented as median (interquartile range). *chi-square test; #Wilcoxon signed-rank test.

Variables	Proportion (n = 303)	GO without Eyelid Edema (n = 144)	GO with Eyelid Edema (n = 159)	p-value
Sex: Female*	157 (51.8%)	75 (52.1%)	82 (51.6%)	0.999
Age, years^#^	47.0 (37.0-54.0)	45.0 (35.0-54.0)	48.0 (41.0-54.5)	0.182
WBC (G/L)^#^	5.9 (4.8-7.6)	5.8 (4.9-7.5)	6.0 (4.7-8.0)	0.479
RBC (T/L)^#^	4.4 (4.2-4.9)	4.4 (4.1-4.9)	4.5 (4.2-4.8)	0.769
Hb (g/L)^#^	135.0 (126.0-148.0)	137.0 (125.0-147.0)	134.0 (126.0-148.0)	0.687
MCV (fL)	90.9 (87.1-94.0)	90.6 (87.7-93.5)	91.1 (86.8-94.4)	0.878
MCHC (g/L)	335.0 (329.0-341.0)	336.5 (328.8-341.0)	334.0 (329.0-340.5)	0.915
Platelets (G/L)^#^	210.0 (173.0-246.0)	216.0 (178.0-245.5)	204.0 (167.0-246.0)	0.163
Neutrophils (G/L)^#^	3.5 (2.7-4.8)	3.5 (2.7-4.4)	3.6 (2.6-5.2)	0.378
Lymphocytes (G/L)^#^	1.7 (1.4-2.1)	1.7 (1.5-2.2)	1.7 (1.3-2.0)	0.034
Monocytes (G/L)^#^	0.4 (0.3-0.5)	0.4 (0.3-0.5)	0.4 (0.3-0.5)	0.321
Eosinophils (G/L)^#^	0.1 (0.0-0.1)	0.1 (0.1-0.2)	0.1 (0.0-0.1)	0.01
Basophils (G/L)^#^	0.0 (0.0-0.0)	0.0 (0.0-0.0)	0.0 (0.0-0.0)	0.687
TBIL (μmol/L)^#^	11.5 (9.1-15.5)	11.6 (9.4-15.6)	11.5 (8.8-15.3)	0.798
ALT (U/L)^#^	20.0 (14.0-29.0)	21.0 (14.5-31.0)	19.0 (13.0-27.0)	0.033
AST (U/L)^#^	19.0 (16.0-23.0)	20.0 (16.5-24.5)	18.0 (15.2-22.0)	0.032
AKP (U/L)^#^	70.0 (57.2-86.0)	69.5 (54.8-84.2)	70.5 (59.2-88.0)	0.25
GGT (U/L)^#^	17.0 (12.0-27.0)	17.0 (13.0-27.0)	16.5 (12.0-27.0)	0.308
Na (mmol/L)^#^	141.1 (139.8-142.7)	141.2 (139.8-142.8)	141.0 (139.8-142.6)	0.728
Total protein^#^	66.3 (62.4-70.0)	66.9 (64.1-70.3)	65.0 (61.5-69.3)	0.013
K (mmol/L)^#^	4.0 (3.8-4.2)	4.0 (3.8-4.2)	3.9 (3.7-4.2)	0.058
Albumin (g/L)^#^	41.3 (39.1-44.5)	41.4 (39.4-44.7)	41.2 (38.9-44.2)	0.412
Cl (mmol/L)^#^	103.2 (101.6-104.7)	103.2 (101.7-105.0)	103.2 (101.5-104.5)	0.448
Ca (mmol/L)^#^	2.2 (2.2-2.3)	2.2 (2.2-2.3)	2.2 (2.2-2.3)	0.624
Uric acid (μmol/L)^#^	295.1 (229.7-360.6)	305.6 (235.8-371.9)	289.4 (227.3-350.5)	0.179
FT3	4.00 (3.55-4.50)	4.10 (3.68-4.53)	3.90 (3.50-4.50)	0.165
FT4	12.70 (11.60-14.00)	13.05 (11.88-14.03)	12.50 (11.30-13.95)	0.131
TSH	1.03 (0.14-2.24)	1.24 (0.26-2.79)	0.59 (0.08-2.19)	0.108
Thyroid function: Not euthyroid	248 (82.4%)	119 (83.8%)	129 (81.1%)	0.649

In addition to key variables, we also analyzed other lab parameters like blood cell counts, liver function tests, and electrolyte levels. The detailed results are in Table 2 for a more complete view of patients' health and systemic factors linked to eyelid edema.

Independent associations of serum ALT and AST levels with eyelid edema

Univariate logistic regression revealed that lower ALT and AST levels were significantly associated with a higher likelihood of eyelid edema (ALT: OR=0.98, 95% CI=0.96-1.00, p=0.0175; AST: OR=0.96, 95% CI=0.93-1.00, p=0.0255) (Table 3). Lymphocyte count also showed a negative correlation (OR=0.59, p=0.0114), while total protein and neutrophils were non-significant. 

**Table 3:** Univariate logistic regression analysis of clinical and laboratory parameters for eyelid edema. WBC: white blood cell; RBC: red blood cell; Hb: hemoglobin; MCV: mean corpuscular volume; MCHC: mean corpuscular hemoglobin concentration; TBIL: total bilirubin; ALT: alanine aminotransferase; AST: aspartate aminotransferase; AKP: alkaline phosphatase; GGT: glutamyl transpeptidase. Results are presented as odds ratios (OR) with 95% confidence intervals (CI).

Variables	OR (95% CI)	p-value
Sex: Female	0.98 (0.62, 1.54)	0.9292
Age	1.01 (0.99, 1.03)	0.2161
Thyroid function: Not euthyroid	0.83 (0.46, 1.51)	0.5440
WBC (G/L)	1.06 (0.97, 1.16)	0.1865
RBC (T/L)	0.92 (0.60, 1.42)	0.7013
Hb (g/L)	1.00 (0.98, 1.01)	0.6857
MCV (fL)	1.00 (0.96, 1.04)	0.9147
MCHC (g/L)	1.00 (0.98, 1.03)	0.7349
Platelets (G/L)	1.00 (0.99, 1.00)	0.1681
Neutrophils (G/L)	1.08 (0.98, 1.19)	0.1056
Lymphocytes (G/L)	0.59 (0.39, 0.89)	0.0114
Monocytes (G/L)	0.77 (0.22, 2.70)	0.6845
Eosinophils (G/L)	1.38 (0.36, 5.22)	0.6352
Basophils (G/L)	0.16 (0.00, 340.28)	0.6406
TBIL (μmol/L)	0.98 (0.94, 1.03)	0.5147
ALT (U/L)	0.98 (0.96, 1.00)	0.0175
AST (U/L)	0.96 (0.93, 1.00)	0.0255
AKP (U/L)	1.00 (1.00, 1.01)	0.2303
GGT (U/L)	0.99 (0.98, 1.00)	0.1008
Na (mmol/L)	1.00 (0.90, 1.10)	0.9528
Total protein	0.97 (0.93, 1.00)	0.0729
K (mmol/L)	0.54 (0.25, 1.17)	0.1171
Albumin (g/L)	0.97 (0.92, 1.04)	0.4004
Cl (mmol/L)	0.97 (0.88, 1.06)	0.4724
Ca (mmol/L)	1.81 (0.23, 14.08)	0.5712
Uric acid (μmol/L)	1.00 (1.00, 1.00)	0.2265

The independent association between ALT and AST with eyelid edema was further validated through multivariable logistic regression (Table 4). After scaling ALT and AST to 10 U/L increments for enhanced clinical interpretability, every 10 U/L decrease in ALT was associated with an 18.7% increased prevalence of eyelid edema (OR=0.813, 95% CI=0.678-0.975), while a similar decrease in AST corresponded to a 31.9% increased prevalence (OR=0.681, 95% CI=0.489-0.947). These associations remained significant after adjusting for sex, age, and lymphocytes, suggesting a potential dose-response relationship between lower liver enzyme levels and higher edema prevalence.

**Table 4:** Adjusted odds ratios for the association between ALT and AST with eyelid edema †Model I: Adjusted for sex and age; ‡Model II: Adjusted for sex, age, and lymphocytes. Results are presented as odds ratios (OR) with 95% confidence intervals (CI).

Variable	Non-adjusted Model	Model I†	Model II‡
ALT (per 10 U/L)	0.814 (0.688, 0.965)	0.805 (0.674, 0.960)	0.813 (0.678, 0.975)
AST (per 10 U/L)	0.696 (0.507, 0.957)	0.681 (0.493, 0.941)	0.681 (0.489, 0.947)

**Figure 1:**
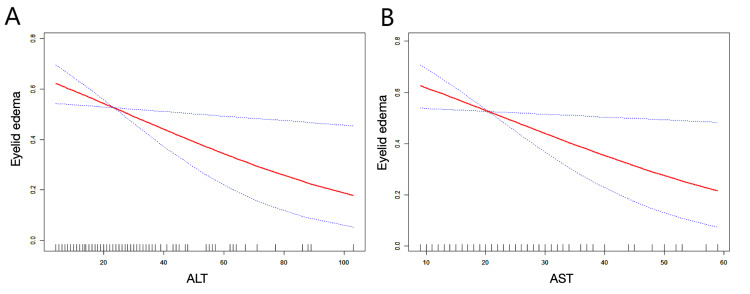
Linear relationship between ALT, AST, and eyelid edema in patients with Graves’ orbitopathy. (A) The relationship between ALT levels and the probability of eyelid edema, showing an inverse association. (B) The relationship between AST levels and the probability of eyelid edema, also showing an inverse association.

To further verify whether there exists a non-linear relationship between AST and ALT with eyelid edema, we performed curve-fitting analysis. The results indicated that there was a linear relationship between ALT and AST levels with the presence of eyelid edema (as shown in Figure 1). This finding suggests that lower concentrations of liver enzymes were associated with an increased prevalence of eyelid edema.

Subgroup analyses

Subgroup analyses were conducted to assess the association between eyelid edema and covariates across different subgroups, including gender, age, and thyroid function status. Consistent trends were observed across different subgroups of sex, age, and thyroid function status, with no significant interactions detected. The ORs and 95% CIs for both ALT and AST were similar within each subgroup, indicating that the main effects were robust and not significantly altered by these covariates (Table 5).

**Table 5:** Subgroup analysis of the association between eyelid edema and covariates.

Variables	N	ALT OR (95% CI)	p	AST OR (95% CI)	p-value
Sex: Male	146	0.97 (0.95, 1.00)	0.0239	0.94 (0.89, 0.99)	0.0116
Sex: Female	157	0.99 (0.96, 1.01)	0.2883	0.99 (0.95, 1.03)	0.5742
Age group (21 -41y)	100	0.96 (0.93, 0.99)	0.0207	0.92 (0.85, 0.99)	0.0293
Age group (42 -51y)	96	0.99 (0.97, 1.02)	0.5252	0.96 (0.90, 1.02)	0.1489
Age group (52 -80y)	107	0.99 (0.95, 1.02)	0.3641	0.99 (0.95, 1.04)	0.7693
Thyroid function: Euthyroid	53	0.97 (0.93, 1.03)	0.3268	0.96 (0.87, 1.06)	0.451
Thyroid function: Not euthyroid	248	0.98 (0.96, 1.00)	0.0254	0.96 (0.93, 1.00)	0.0327

## Discussion

Inverse association of serum ALT and AST levels with eyelid edema in GO

This study demonstrates a significant inverse association between serum ALT and AST levels with eyelid edema in patients with GO, which persisted after adjusting for age, sex, and other laboratory parameters. These findings suggest a potential role of liver function in the pathophysiology of GO-related edema, warranting further investigation into the underlying mechanisms.

Potential mechanisms underlying the ALT/AST-edema association

Untreated Graves' disease patients frequently exhibit hepatic abnormalities, with 60% showing ≥1 abnormal liver parameter, including abnormal ALT (33%) and AST (23%) [11]. The association between lower serum ALT and AST levels and eyelid edema in GO may be explained through multifactorial mechanisms involving systemic metabolic alterations, inflammatory pathways, and tissue remodeling. Although ALT and AST are traditionally recognized as biomarkers of hepatocellular injury, their reduced levels in this context could reflect broader systemic changes linked to thyroid dysfunction, autoimmunity, or altered metabolic states.

First, thyroid hormone dysregulation in Graves’ disease may influence hepatic metabolism and enzyme synthesis [13]. Hyperthyroidism accelerates metabolic processes, potentially leading to increased catabolism of proteins, including hepatic enzymes [14-16]. Reduced ALT and AST levels could indicate suppressed hepatic synthetic function due to hypermetabolic stress or nutrient depletion, particularly in cases of prolonged thyrotoxicosis [17,18]. Concurrently, hypoalbuminemia, a potential consequence of hyperthyroidism-induced protein catabolism, may exacerbate vascular permeability, contributing to fluid accumulation in periocular tissues [19].

Second, systemic inflammation in GO may modulate ALT and AST dynamics. Chronic inflammation in autoimmune thyroid disease is characterized by elevated pro-inflammatory cytokines (e.g., IL-6, TNF-α), which can suppress hepatic enzyme production or alter their release kinetics [20]. Lower ALT and AST levels might reflect a compensatory mechanism to mitigate oxidative stress, as these enzymes are also involved in glutathione metabolism and redox balance [21]. Conversely, localized inflammation in orbital tissues could drive edema through cytokine-mediated vascular leakage, independent of hepatic enzyme activity [20].

Third, tissue-specific interactions between thyroid autoimmunity and skeletal muscle or adipose tissue may play a role. AST is expressed in extrahepatic tissues, including skeletal muscle, and its reduction could signal muscle wasting or metabolic adaptation in GD [18]. Eyelid edema, a hallmark of GO, is driven by glycosaminoglycan deposition, adipogenesis, and lymphatic dysfunction, processes regulated by insulin-like growth factor 1 (IGF-1) and TSHR-Ab. Interestingly, hepatic IGF-1 synthesis is thyroid hormone-dependent, and hypoalbuminemia may further impair fluid homeostasis, linking hepatic function to orbital pathology [22,23].

Lastly, mitochondrial dysfunction in hyperthyroidism could reduce cellular energy production, affecting both hepatocyte viability (lower enzyme release) and orbital fibroblast activity. Impaired mitochondrial respiration may exacerbate oxidative damage in orbital tissues, promoting edema through reactive oxygen species (ROS)-mediated mechanisms [24,25].

In summary, lower ALT and AST levels in GO may reflect systemic metabolic stress, inflammation-driven suppression of hepatic enzymes, or tissue-specific crosstalk between thyroid autoimmunity and orbital remodeling. Exploring these associations offers several clinical and therapeutic implications. First, ALT and AST could serve as easily accessible and cost-effective biomarkers for assessing disease activity and severity in GO, complementing existing markers like TSHR-Ab [12]. Their widespread availability in routine clinical practice could facilitate more frequent monitoring of patients. Second, identifying a potential link between liver enzyme levels and orbital inflammation might provide insights into the systemic nature of GO, guiding the development of more targeted therapeutic interventions. For example, understanding the metabolic and inflammatory pathways involved could lead to novel treatment approaches aimed at modulating these pathways. Lastly, incorporating liver enzyme levels into the clinical assessment of GO patients might enhance the prediction of disease progression and response to treatment, allowing for more personalized therapeutic strategies. Further studies are needed to clarify causal pathways and fully explore the therapeutic implications of these associations.

Comparative evidence from other autoimmune diseases

The inverse ALT/AST-edema relationship mirrors findings in systemic lupus erythematosus (SLE), where lower liver enzymes correlate with serositis, possibly due to hepatic sequestration of inflammatory mediators [26,27]. Similarly, in rheumatoid arthritis, methotrexate-induced ALT reduction coincides with decreased synovial inflammation [28]. These parallels underscore the potential role of liver enzymes as systemic inflammation sentinels in GO.

Limitations

Our study has several limitations that should be acknowledged. First, as a cross-sectional study, our research cannot establish a causal relationship between serum ALT and AST levels with eyelid edema. Cohort studies are needed to clarify whether lower liver enzyme levels precede the development of edema or are a consequence of systemic changes associated with GO. Second, despite adjusting for key covariates, residual confounding from unmeasured factors (e.g., subclinical hepatic steatosis, smoking history, alcohol intake, or dietary habits) may have influenced our results. Future studies incorporating detailed clinical and lifestyle data are warranted to address this issue. Third, the exclusion of patients on anti-thyroid medications may limit the generalizability of our findings to the broader GO population, particularly those with active thyroid dysfunction. However, this exclusion was necessary to minimize the potential confounding effects of these drugs on liver enzyme levels and systemic inflammation.

## Conclusions

Lower serum ALT and AST levels are independently associated with eyelid edema in patients with GO, even after adjusting for age, sex, and lymphocytes. These findings suggest a potential link between liver function and the pathogenesis of GO-related edema, highlighting the need for further investigation into the underlying mechanisms. Future longitudinal studies are warranted to establish causality and explore the clinical utility of liver enzymes as biomarkers for GO severity or progression.
